# Dendritic cell-targeting chemokines inhibit colorectal cancer progression

**DOI:** 10.37349/etat.2022.00115

**Published:** 2022-12-27

**Authors:** Pengkun Yuan, Yunyi Zhou, Zhixue Wang, Liming Gui, Bin Ma

**Affiliations:** 1 School of Biomedical Engineering, Med-X Research Institute, Shanghai Jiao Tong University, Shanghai 200030, China; 2 Clinical Stem Cell Research Center, Renji Hospital, School of Medicine, Shanghai Jiao Tong University, Shanghai 200127, China; 3 Zhejiang University–University of Edinburgh (ZJU-UoE) Institute, Zhejiang University School of Medicine, Hangzhou 310058, Zhejiang, China; Université Paris-Saclay, France

**Keywords:** Dendritic cells, chemokines, tumor microenvironment, immunotherapy, colorectal cancer

## Abstract

**Aim:**

Recent progress in cancer immunotherapy has shown its promise and prompted researchers to develop novel therapeutic strategies. Dendritic cells (DCs) are professional antigen-presenting cells crucial for initiating adaptive anti-tumor immunity, therefore a promising target for cancer treatment. Here, anti-tumor activities of DC-targeting chemokines were explored in murine colorectal tumor models.

**Methods:**

The correlation of chemokine messenger RNA (mRNA) expression with DC markers was analyzed using The Cancer Genome Atlas (TCGA) dataset. Murine colorectal tumor cell lines (CT26 and MC38) stably overexpressing mouse C-C motif chemokine ligand 3 (CCL3), CCL19, CCL21, and X-C motif chemokine ligand 1 (XCL1) were established by lentiviral transduction. The effect of chemokines on tumor cell proliferation/survival was evaluated *in vitro* by cell counting kit-8 (CCK-8) assay and colony formation assay. Syngeneic subcutaneous tumor models were used to study the effects of these chemokines on tumor growth. Ki-67 expression in tumors was examined by immunohistochemistry. Immune cells in the tumor microenvironment (TME) and lymph nodes were analyzed by flow cytometry.

**Results:**

Expression of the four chemokines was positively correlated with the two DC markers [integrin alpha X (*ITGAX*) and *CLEC9A*] in human colorectal tumor samples. Tumoral overexpression of DC-targeting chemokines had little or no effect on tumor cell proliferation/survival *in vitro* while significantly suppressing tumor growth *in vivo*. Fluorescence-activated cell sorting (FACS) analysis showed that CCL19, CCL21, and XCL1 boosted the ratios of DCs and T cells in CD45^+^ leukocytes while CCL3 increased the percentage of CD45^+^ leukocytes in total cells in MC38 tumor. XCL1 had an additional positive effect on antigen uptake by DCs in the TME and antigen transfer to tumor-draining lymph nodes.

**Conclusions:**

CCL3, CCL19, CCL21, and XCL1 exhibited potent anti-tumor activities *in vivo*, although they might differentially regulate immune cells in the TME and antigen transfer to lymph nodes.

## Introduction

Colorectal cancer (CRC) is the third most frequently occurring cancer and the second leading cause of cancer death worldwide in 2020 [1]. Despite the rapid development of checkpoint inhibition immunotherapy, it is only effective in a small proportion of CRC patients [2, 3]. Therefore, more immunotherapeutic strategies need to be explored for improved clinical outcomes.

The tumor microenvironment (TME) is one of the primary regulators of CRC development, metastasis, and resistance to therapies [4, 5]. CRC tumors evade immunosurveillance through various extrinsic mechanisms, including repression and exclusion of anti-tumor effector cells such as CD8^+^ T cells and dendritic cells (DCs), and accumulation of immunosuppressive cells in the TME such as regulatory T (Treg) cells and myeloid-derived suppressor cells (MDSCs) [6, 7]. DCs are crucial for inducing adaptive anti-tumor immune responses, especially the tumor-infiltrating conventional DCs (cDCs) [8–11]. Type 1 cDC (cDC1) is regarded as the main subset that can cross-present antigens to activate CD8^+^ T cells through the major histocompatibility complex (MHC)-I pathway, prime CD4^+^ T cells via MHC-II and CD40 signaling, and promote T helper 1 (Th1) and natural killer (NK) cells responses through interleukin (IL)-12 [8–10, 12, 13]. On the other hand, cDC2 can activate Th1, Th2, Th17, and CD8^+^ T cells *in vitro* and drive anti-tumor CD4^+^ T cell immunity [11, 14, 15]. Notably, cDCs are indispensable for adoptive T cell immunotherapy [16]. Therefore, efforts to increase cDCs in the TME would serve as an alternative therapeutic strategy and improve other immunotherapies’ efficacies.

CD11c, encoded by the gene integrin alpha X (*ITGAX*), is a widely established marker for DCs. It is often employed to define DCs and their subsets [17, 18], and can be used as a prognostic marker for cancer and other diseases [19]. Human CD141^+^ DCs belong to cDC1, and are considered as the equivalents of the mouse CD8α^+^/CD103^+^ cDCs in functions and lineage [20]. And it is reported that human CD141^+^ DCs and mouse CD8α^+^/CD103^+^ cDCs both express C-type lectin receptor (CLR) CLEC9A which recognizes F-actin exposed by dead cells to mediate the cross-presentation process [21, 22]. CLEC9A has been used as a successful target for antibody-mediated delivery of antigens to cDC1 [23]. Overall, ITGAX and CLEC9A are favorable DC markers for total DC and cDC1 respectively for the analysis of patient samples consisting of various cell types.

Chemokines are important mediators of DC migration and play a significant role in coordinating anti-tumor immunity [24]. Various chemokines have abilities to recruit cDCs, including C-C motif chemokine ligand 3 (CCL3), CCL4, CCL5, CCL19, CCL20, CCL21, and X-C motif chemokine ligand 1 (XCL1) [25, 26]. Although upregulation of these chemokines in the TME is expected to enhance DC-mediated anti-tumor immunity, they may also exert pro-tumor behavior via regulation of tumor cells and other resident cells in the TME such as fibroblasts, endothelial cells, or other pro-tumorigenic immune cells [27–30]. While more evidence supports a pro-tumor role of CCL4, CCL5, and CCL20 [27–30], the effect of others may be context-dependent, indicating the complexity of the underlying interrelated signaling cascades [31, 32]. Therefore, CCL3, CCL19, CCL21, and XCL1 were eventually chosen as the focus of the current study. Despite a few reports suggesting that they participated in pro-tumor activities [31–33], we should not ignore their positive roles in eliciting anti-tumor immunity. In this study, we sought to determine whether those four chemokines could control tumor growth and regulate immune cells in murine models of CRC.

## Materials and methods

### Analysis of human The Cancer Genome Atlas database

The correlation of messenger RNA (mRNA) expression between chemokines and DC markers was analyzed using the cBioPortal (http://www.cbioportal.org/). Colorectal adenocarcinoma dataset of 592 samples with mRNA data (RNA Seq V2) from The Cancer Genome Atlas (TCGA), Pan-Cancer Atlas, was used for the analysis. Expression Z-scores of tumor samples compared to the expression distribution of all log-transformed mRNA expression of adjacent normal samples in the cohort (log RNA Seq V2 RSEM). The Z-score threshold was set at ± 2.0. Analytical methods included both Spearman’s rank correlation and Pearson correlation were calculated, and their correlation coefficients and respective *P* values were shown in the Results.

### Cell lines

CT26 and MC38 cell lines were cultured in Roswell Park Memorial Institute Medium (RPMI 1640) and Dulbecco’s Modified Eagle Medium (DMEM), respectively, supplemented with 10% fetal bovine serum (FBS, Sigma-Aldrich) and 100 U/mL penicillin/streptomycin (Beyotime). Cell lines were authenticated by Shanghai Biowing Applied Biotechnology, and routinely tested for mycoplasma contamination. All cells were maintained in a 5% CO_2_, humidified incubator at 37°C. CT26 and MC38 were infected with CCL3-, CCL19-, CCL21-, and XCL1-overexpressing lentiviruses co-expressing ZsGreen, in the presence of 6 μg/mL polybrene to generate cell lines stably expressing the chemokines or empty vector.

### RNA extraction and quantitative reverse transcription-polymerase chain reaction

Total RNA was isolated by the RNA extraction kit (TIANGEN Biotech), and complementary DNA (cDNA) was synthesized by reverse transcription kit (Vazyme). Real-time polymerase chain reaction (PCR) was carried out using TB Green^®^ *Premix Ex Taq*™ (Takara^®^) in the ABI 7900HT fast real-time PCR system (Applied Biosystems). mRNA expression levels of target genes were normalized to the glyceraldehyde 3-phosphate dehydrogenase (*Gapdh*) using the 2^−ΔΔCt^ method.

### Cell counting kit-8 assay

Proliferation/survival of CT26 and MC38 cells overexpressing chemokines or vector was determined by cell counting kit-8 (CCK-8, TargetMol^®^) and measured by a microplate reader (Tecan Infinite^®^ M Plex) every 24 h according to the protocols from the manufacturer.

### Colony formation assay

Cells were plated at densities of 500, 1,000, and 2,000 cells/well in 12-well plates using a two-layer soft agar setting. The bottom of each well was covered by an agar layer, consisting of 1× DMEM complete medium with 0.6% (*w*/*v*) agarose. Subsequently, different concentrations of cells suspended in 1× DMEM complete medium with 0.35% (*w*/*v*) agarose were added on top of the bottom agar layer. After 10 days, colonies were stained with 0.005% crystal violet solution and counted using ImageJ software.

### Syngeneic mouse models of CRC

Six- to eight-week-old male C57BL/6 mice and male BALB/c mice were purchased from Shanghai Lingchang BioTech Co., Ltd. The 0.5 × 10^6^ CT26 or 1 × 10^6^ MC38 cells overexpressing chemokines or vector were injected subcutaneously at the lower flank of mice in 100 μL phosphate-buffered saline (PBS) respectively. Tumor size was measured by a vernier caliper every three days and calculated as Tumor Volume (mm^3^) = Length (mm) × Width (mm) × Width (mm)/2. Mice were euthanized when tumor size reached 2,000 mm^3^ in volume or the tumor became ulcerated.

### Immunohistochemistry

Whole tumors were fixed for 24 h in 4% formalin and subsequently embedded into paraffin, sectioned, and then mounted onto slides. After antigen retrieval, permeabilization, and blocking, anti-Ki-67 antibody (Abcam, ab16667) was incubated with the epitope at 4°C overnight, followed by the addition of horseradish peroxidase (HRP)-conjugated goat anti-rabbit immunoglobulin G (IgG; Abcam, ab6721, at 1:200 dilution) for another 30 min in dark at room temperature. Next, sections were treated with 3,3’ diaminobenzidine (DAB) substrate (Abcam, ab64238) and imaged by microscope (Leica DM6 B). The ratio of Ki-67^+^ cells was analyzed using ImageJ software. Four different fields were counted to calculate the average ratio for each tumor.

### Flow cytometry

Mice were euthanized via CO_2_ asphyxiation 15 days after the establishment of MC38 tumors. Tumor tissues were excised and cut into 1–2 mm^3^ pieces, and then treated with 1 mg/mL Liberase™ TL (Roche) and 100 mg/mL DNase I (Sigma-Aldrich) in gentleMACS™ Octo Dissociator (Miltenyi Biotec). After dissociation of tumor tissues, cell suspensions were passed through 70-μm cell strainers (BioFil^®^) and washed twice with PBS containing 2% FBS. Inguinal lymph nodes were mechanically dissociated and passed through 70-μm cell strainers (BioFil^®^) to obtain single-cell suspensions.

Before antibody staining, single-cell suspensions were stained with Zombie dyes (BioLegend^®^) at a dilution of 1:200 for 10 min at room temperature to exclude dead cells. Markers on the cell surface were detected by staining cells with fluorochrome-labeled antibodies in PBS containing 2% FBS and 1 mmol/L ethylenediaminetetraacetic acid (EDTA) for 30 min on ice. For subsequent intracellular cytokine analysis, cells were washed once with PBS and treated with the True-Nuclear™ Transcription Factor Buffer Set (BioLegend^®^). The following antibodies were used: CD45 (30-F11, BD Biosciences, dilution 1:200); CD11c (N418, BioLegend^®^, dilution 1:100); CD11b (M1/70, BioLegend^®^, dilution 1:100); CD103 (2E7, BioLegend^®^, dilution 1:100); Ly6C (HK1.4, BioLegend^®^, dilution 1:100); CD64 (X54-5/7.1, BioLegend^®^, dilution 1:100); MHC-II (I-A/I-E, M5/114.15.2, BioLegend^®^, dilution 1:100); CD3e (145-2C11, BioLegend^®^, dilution 1:100); CD4 (GK1.5, BioLegend^®^, dilution 1:100); NK1.1 (PK136, BioLegend^®^, dilution 1:100); CD8a (53-6.7, BioLegend^®^, dilution 1:100); Foxp3 (MF-14, eBioscience, dilution 1:100). The cDCs were defined as: CD45^+^CD11c^+^MHC-II^+^CD64^–^Ly6C^–^; the cDC1: CD45^+^CD11c^+^MHC-II^+^CD64^–^Ly6C^–^CD103^+^; the cDC2: CD45^+^CD11c^+^MHC-II^+^CD64^–^Ly6C^–^CD11b^+^; the macrophages: CD11b^+^CD64^+^Ly6C^–^; the conventional CD4^+^ T cells: CD45^+^CD3e^+^CD4^+^Foxp3^–^; the Treg cells: CD45^+^CD3e^+^CD4^+^Foxp3^+^; the CD8^+^ T cells: CD45^+^CD3e^+^CD8^+^; the NK cells: CD45^+^CD3e^–^NK1.1^+^; the NKT cells: CD45^+^CD3e^+^NK1.1^+^. Fluorescence data were acquired on a BD LSRFortessa™ cell analyzer (BD Biosciences). Data were analyzed on FlowJo™ software.

### Statistical analysis

Statistical tests were performed in GraphPad Prism 8. CCK-8 assay, colony formation assay, immunohistochemistry, and tumor growth *in vivo* were analyzed via two-way analysis of variance (ANOVA) followed by multiple comparisons; data from flow cytometry were analyzed via one-way ANOVA followed by multiple comparisons. Results were presented as mean values ± standard error of the measurement (SEM), and *P* < 0.05 was considered statistically significant.

## Results

### Expression of *CCL3*, *CCL19*, *CCL21*, and *XCL1* correlates with DC markers in human CRC patient samples

**Figure 1.**
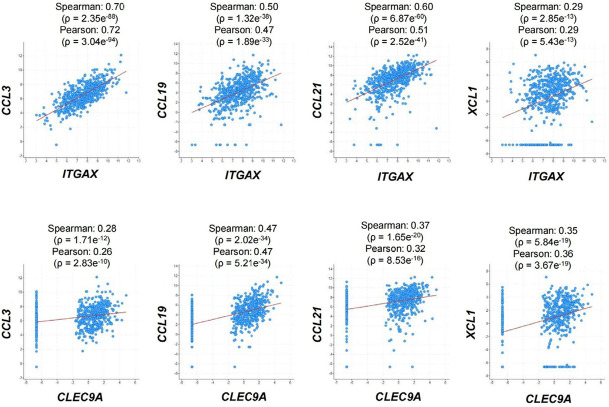
Correlation of mRNA expression between chemokines and DC markers. RNA-sequencing dataset from 592 colorectal adenocarcinoma patients (TCGA, Pan-Cancer Atlas) was used to analyze the expression correlation between *CCL3*/*CCL19*/*CCL21*/*XCL1* and *ITGAX*/*CLEC9A*. Dot plots indicate mRNA expression levels in tumor samples relative to normal samples (Z-score, log_2_ RNA Seq V2 RSEM)

To explore the potential *in vivo* role of the selected chemokines in regulating DCs in human CRC patients, we first analyzed the correlation of their mRNA expression with DC markers. Expression of all the four chemokine genes, including *CCL3*, *CCL19*, *CCL21*, and *XCL1*, was positively correlated with that of both *ITGAX* encoding the general DC marker CD11c and *CLEC9A*, which is a cDC1 marker, in human CRC samples (Figure 1) [34]. These findings implied that all the four chemokines played a positive role in DC infiltration in human colorectal tumors, although they might have discrepant activities in regulating different DC subsets and other immune cells.

### Tumoral overexpression of DC-targeting chemokines showed no or little effects on tumor cell proliferation/survival *in vitro*

**Figure 2.**
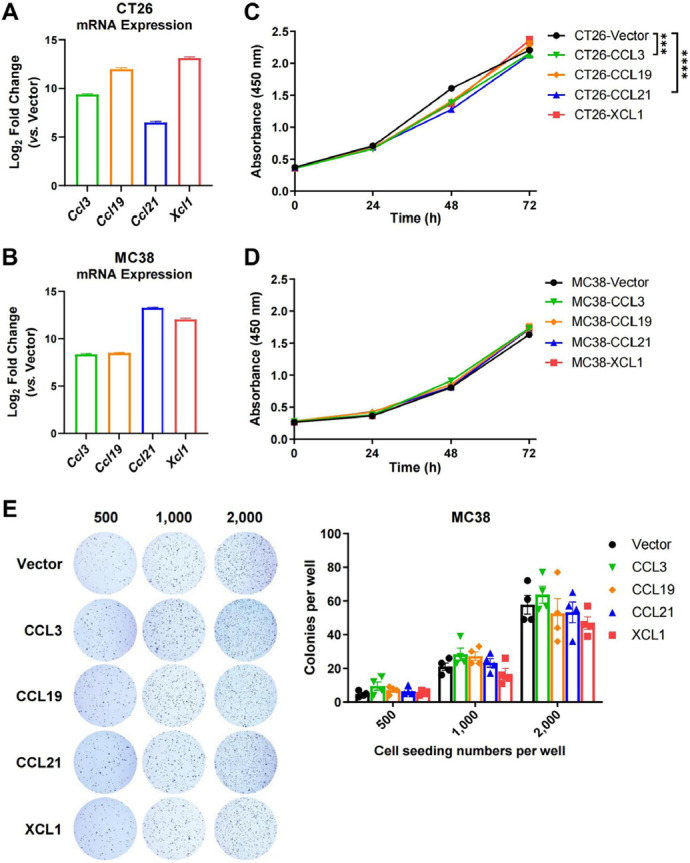
Chemokine overexpression hardly affects tumor cell proliferation or survival. A. and B. The expression of mouse chemokine genes (*Ccl3*/*Ccl19*/*Ccl21*/*Xcl1*) in CT26 (A) and MC38 (B) cell lines was confirmed by quantitative PCR (qPCR, *n* = 4–5); C. and D. CCK-8 assay was used to quantify the amount of CT26 (C, *n* = 3) and MC38 cells (D, *n* = 5) overexpressing chemokines or vector over a 72-h culture period; E. representative images for colony formation assay and statistics of colony numbers of MC38 overexpressing chemokines or empty vector at indicated seeding densities (*n* = 4). ^***^ *P* < 0.001; ^****^ *P* < 0.0001

To clarify the anti- or pro-tumor effects of the DC-targeting chemokines, we generated murine CRC cell lines that stably expressed the four chemokines. Successful overexpression of mouse CCL3, CCL19, CCL21, and XCL1 (encoded by *Ccl3*/*Ccl19*/*Ccl21*/*Xcl1*) in CT26 and MC38 cell lines was confirmed by quantitative reverse transcription-PCR (Figure 2A and 2B). CCK-8 assay demonstrated that overexpression of these genes did not significantly change the total numbers of cells during the 72-h culture period, compared to empty vector control, except that CCL3 and CCL21 reduced the growth rate of CT26 cells but to a limited extent (Figure 2C and 2D). Moreover, the colony-forming ability of MC38 cells was not affected by these chemokines (Figure 2E). It suggested that the chemokines had trivial or no direct effect on the proliferation or survival of tumor cells.

### Tumoral overexpression of DC-targeting chemokines inhibits tumor growth in mice

**Figure 3.**
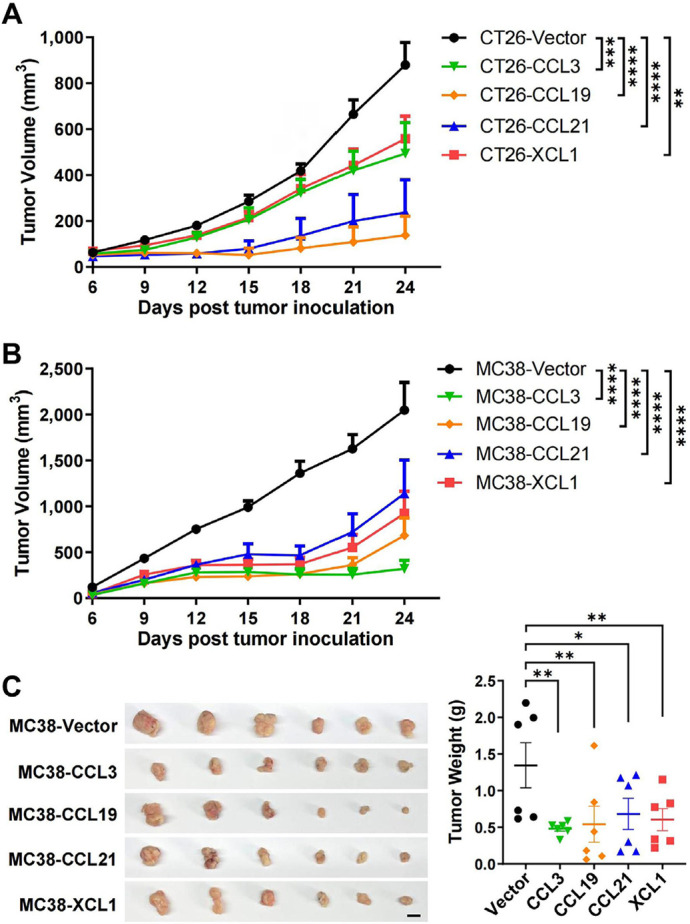
Tumoral overexpression of chemokines inhibits colorectal tumor growth. Volumes of CT26 tumors (*n* = 5/group, A) or MC38 tumors (*n* = 8/group, B) overexpressing chemokines or vector were monitored overtime; C. images and weights of MC38 tumors, scale bar = 1 cm. ^*^ *P* < 0.05; ^**^ *P* < 0.01; ^***^ *P* < 0.001; ^****^ *P* < 0.0001

**Figure 4.**
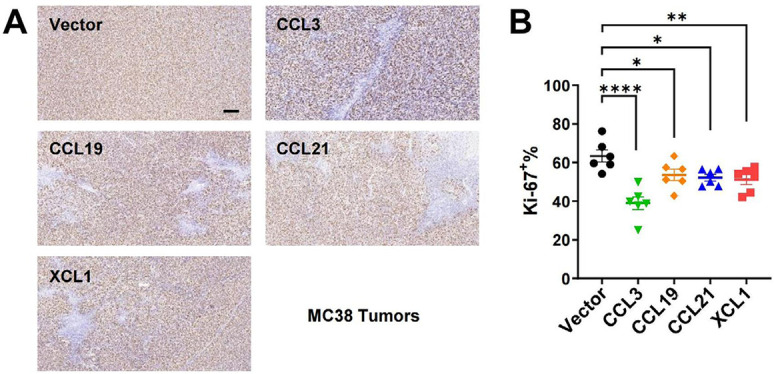
Immunohistochemistry for Ki-67 in MC38 tumors. A. Representative images of Ki-67 staining, scale bar = 50 μm; B. statistics of Ki-67^+^ ratios (*n* = 6/group). ^*^ *P* < 0.05; ^**^ *P* < 0.01; ^****^ *P* < 0.0001

We then established subcutaneous tumor models using CT26 and MC38 overexpressing the four chemokines. In both models, tumoral overexpression of CCL3, CCL19, CCL21, and XCL1 significantly repressed tumor growth (Figure 3A–C), despite the differential overexpression levels in these cell lines (Figure 2A and 2B). Consistently, these chemokines also decreased the ratio of Ki-67^+^ cells in MC38 tumors indicating a negative effect on tumor cell growth *in vivo* (Figure 4A and 4B). Taking the results from CT26 and MC38 models together, CCL19 seemed to be the most potent anti-tumor chemokine among the four. Since these chemokines had no or little effects on the proliferation or survival of tumor cells (Figure 2C and 2D), their anti-tumor activities *in vivo* were very likely to depend on their immunomodulatory effects in the TME.

### Regulation of DCs in MC38 tumors and lymph nodes by chemokines

**Figure 5.**
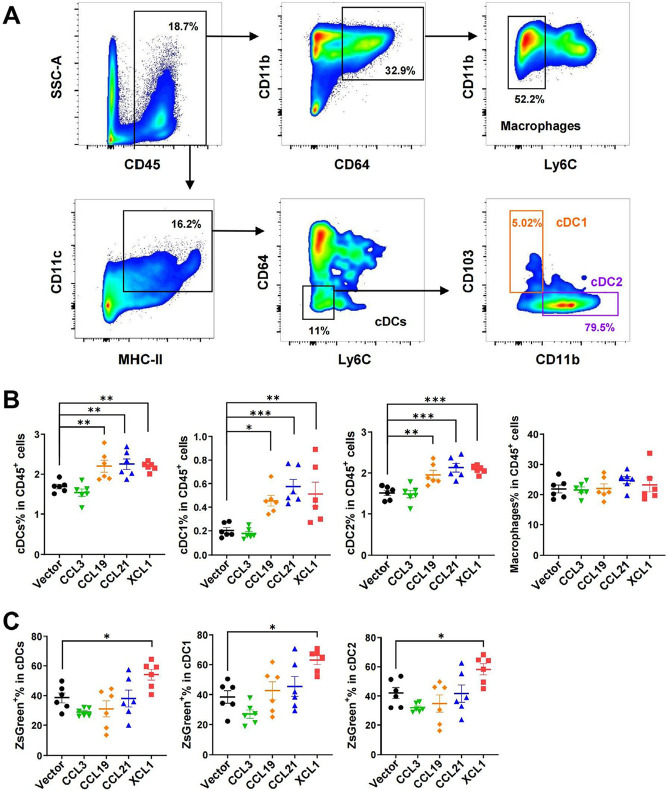
FACS analysis of tumor-infiltrating cDCs and macrophages in MC38 tumor model. A. Gating strategies for cDCs, cDC1, cDC2, and macrophages; B. ratios of cDCs, cDC1, cDC2, and macrophages in CD45^+^ cells; C. ratios of ZsGreen^+^ cells in cDCs. ^*^ *P* < 0.05; ^**^ *P* < 0.01; ^***^ *P* < 0.001; SSC-A: side scatter-area

Next, we sought to investigate the effects of these chemokines on DC migration in MC38 tumor models by fluorescence-activated cell sorting (FACS). MC38 cells expressed ZsGreen simultaneously as an indicator of tumor-specific antigen. Tumoral overexpression of CCL19, CCL21, and XCL1 significantly increased ratios of total cDCs, cDC1, and cDC2 subtypes among CD45^+^ leukocytes in the TME, as compared to the empty vector control tumors (Figure 5A and 5B). However, CCL3 did not change the ratios of total cDCs or the two subtypes. In contrast, the percentages of macrophages were not affected by either chemokine, suggesting these chemokines were DC-specific. In particular, the ratio of ZsGreen^+^ cDCs was upregulated in XCL1-overexpressing tumors, suggesting an enhanced uptake of tumor antigens (Figure 5C).

**Figure 6.**
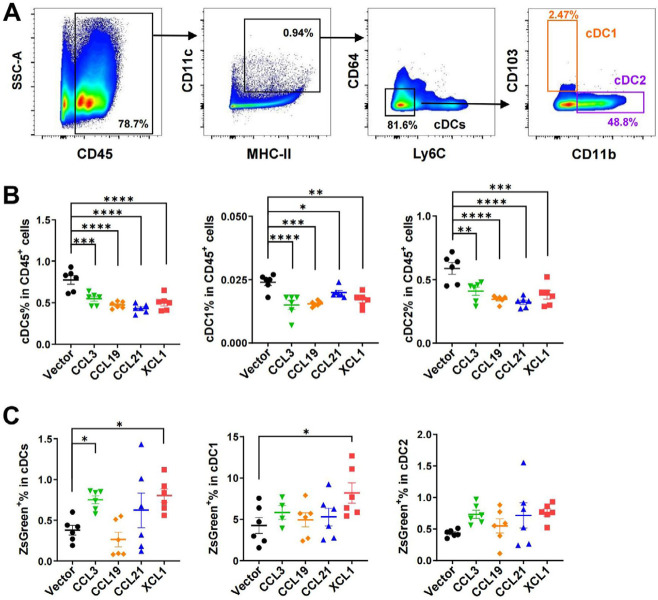
FACS analysis of cDCs in tumor-draining lymph nodes in MC38 tumor model. A. Gating strategies for cDCs, cDC1, and cDC2; B. ratios of cDCs, cDC1, and cDC2 in CD45^+^ cells; C. ratios of ZsGreen^+^ cells in cDCs. ^*^ *P* < 0.05; ^**^ *P* < 0.01; ^***^ *P* < 0.001; ^****^ *P* < 0.0001

Interestingly, cDCs, including cDC1 and cDC2 in the tumor-draining lymph nodes, were significantly decreased by tumoral expression of these chemokines, implying an efflux of cDCs from the lymph nodes or a biased upregulation of other immune cells such as T cells upon antigen presentation and stimulation (Figure 6A and 6B). Higher percentages of ZsGreen^+^ cDCs were found in the lymph nodes of CCL3- and XCL1-overexpressing tumor models (Figure 6C). This finding suggested that more tumor antigens were delivered by cDCs from tumors overexpressing these two chemokines, and possibly more tumor-specific T cells would be generated. The increased tumor antigen ZsGreen in the lymph nodes of XCL1-overexpressing tumor models may be partially attributed to the increased antigen uptake in the tumor (Figure 5C). These data demonstrated that tumoral expression of these chemokines did not impair or even promote the antigen transfer from the tumor site to tumor-draining lymph nodes.

### Regulation of tumor-infiltrating lymphocytes by chemokines

**Figure 7.**
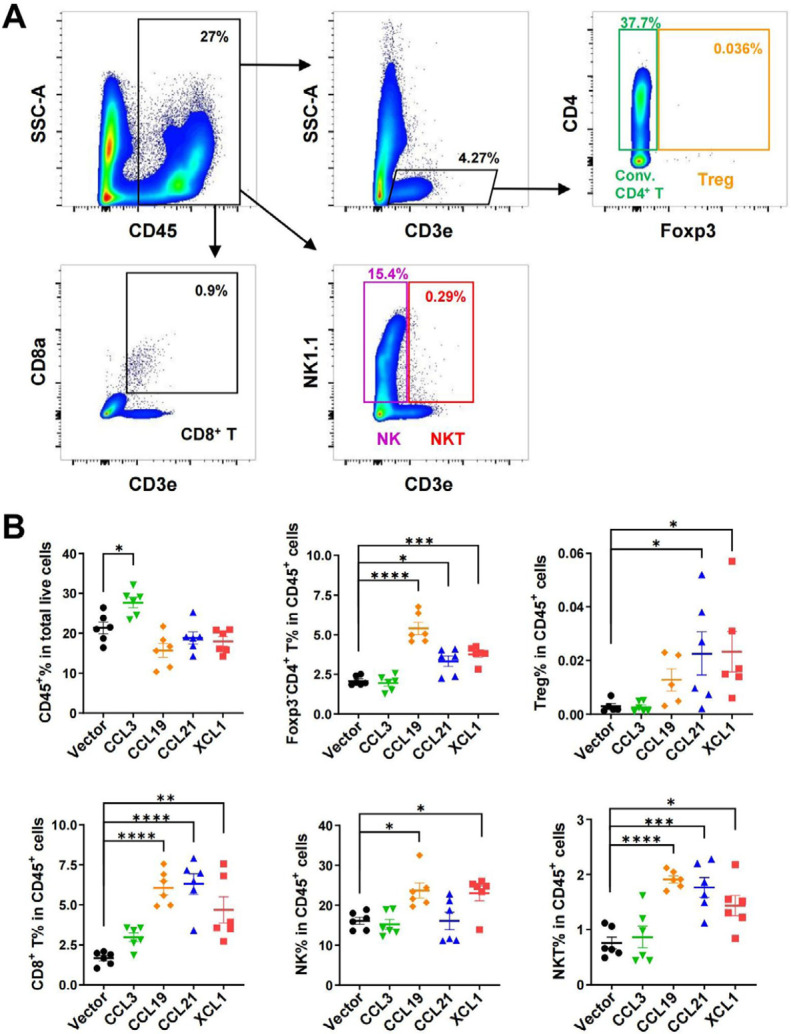
FACS analysis of tumor-infiltrating lymphocytes in MC38 tumor model. A. Gating strategies for conventional (conv.) CD4^+^ T, Treg, CD8^+^ T, NK, and NKT cells; B. ratio of CD45^+^ cell in total live cells, and ratios of lymphocytes in CD45^+^ cells. ^*^ *P* < 0.05; ^**^ *P* < 0.01; ^***^ *P* < 0.001; ^****^ *P* < 0.0001

Confirming a positive effect on cDCs in TME and lymph nodes, we further analyzed the tumor-infiltrating effector lymphocytes by FACS (Figure 7A). CD45^+^ leukocytes in total live cells were upregulated only in CCL3-overexpressing tumors, although the ratios of lymphocytes in CD45^+^ cells were not changed by CCL3 (Figure 7B). Consistent with their effects on cDCs, CCL19, CCL21, and XCL1 significantly increased the ratios of conventional Foxp3^–^CD4^+^ T and CD8^+^ T cells. These three chemokines also elevated ratios of Treg cells, although the effect of CCL19 was not statistically significant. NK cells were upregulated by CCL19 and XCL1, while CCL19, CCL21, and XCL1 upregulated NKT. Together, these results revealed that these chemokines boosted anti-tumor adaptive immunity and enrichment of other effector cells with anti-tumor activities such as NK and NKT cells.

## Discussion

In agreement with their anti-tumor potentials, a higher density of tumor-infiltrating cDCs is associated with a better prognosis for patients with various tumor types [35–37]. Here we evaluated the anti-tumor activities of several DC-targeting chemokines in parallel to explore the possibility of using them for immunotherapy. We focused on the chemokines with less evidence of a pro-tumor role. In mouse CRC models, we demonstrated that all four chemokines, including CCL3, CCL19, CCL21, and XCL1, effectively inhibited tumor growth. Since these chemokines showed hardly any effect on the proliferation/survival of tumor cells *in vitro*, their functions *in vivo* would largely depend on the regulation of TME cells. Accordingly, they significantly increased the ratios of cDCs and T cells in CD45^+^ cells in the MC38 tumor, except CCL3 (Figures 5 and 7). However, CCL3 was the only chemokine that upregulated the percentage of CD45^+^ cells out of total cells from the tumor (Figure 7B), suggesting it might have a broad impact on a variety of leukocytes leading to an unbiased ratio of cDCs or T cells in CD45^+^ cells. Indeed, CCL3 has a complex role in mediating a great range of cell types via three distinct receptors, including C-C motif chemokine receptor 1 (CCR1), CCR4, and CCR5 [25, 38]. Interestingly, our FACS analysis indicated that CCL3 promoted the transfer of tumor antigens to lymph nodes (Figure 6C). As a result, it can be speculated that more tumor-specific T cells will appear, contributing to the anti-tumor activity of CCL3.

Both CCL19 and CCL21 signal through CCR7 and are essential to building an effective interaction between T cells and DCs in lymphoid tissues [25, 38]. Differing from CCL19, CCL21 has a long C-terminal tail with 37 amino acids and is competent to interact with glycosaminoglycans (GAGs) and then immobilizes the chemokine [39, 40], which may explain the differential activities of these two chemokines [41]. CCL21 was shown to be superior to CCL19 in promoting chimeric antigen receptor (CAR)-T activity [41]. In both CT26 and MC38 tumor models of our study, CCL19 and CCL21 showed powerful anti-tumor activities, with CCL19 being more potent than CCL21 (Figure 3), although the overexpression levels varied in the two tumor cell lines. In addition to a role in recruiting cDCs, CCL19 and CCL21 may also induce the proinflammatory differentiation program in DCs [42]. Moreover, they may regulate migration and proliferation/survival of T cells [43, 44]. The other mechanisms besides DC recruitment added to the complexity of their anti-tumor actions. Accordingly, in MC38 tumors, conventional CD4^+^ T and CD8^+^ T cells were boosted by these two chemokines. Notably, CCL19 overexpression induced higher ratios of tumor-infiltrating conventional CD4^+^ T and NK cells but fewer Treg cells compared to CCL21, which may partially contribute to the more prominent anti-tumor effect of CCL19. Nevertheless, both seem to be promising immunotherapeutic molecules, and more context-dependent studies are needed to select the optimal one.

Consistent with the previous report that XCL1 was able to attract CD103^+^ DCs in murine models [45, 46], XCL1 was found to increase CD103^+^ cDC1 and CD11b^+^ cDC2 in MC38 tumors in our study (Figure 5B). Furthermore, our findings also revealed an unexpected role of XCL1 in enhancing tumor antigen uptake by cDCs and antigen transfer to lymph nodes (Figure 5C and 6C). Whether the regulation of DC function by XCL1 is via direct activation of X-C motif chemokine receptor 1 (XCR1) on DCs remains to be elucidated.

The major limitation of the current study is that the overexpression levels of these chemokines were not equal in tumor cells due to technical difficulties. Thus, the study of these chemokines by tumoral overexpression is relatively qualitative, and a comparison of their activities is somewhat preliminary and suggestive. However, CCL19 seemed to be the most potent anti-tumor chemokine among the four if considering results from both CT26 and MC38 models. Nevertheless, our study still provides the foundation for prompting these chemokines into more sophisticated preclinical studies.

A variety of DC-based immunotherapeutic strategies are being perused in preclinical and clinical studies [34]. The delivery of DC-targeting chemokines to the tumor site represents an ideal way to boost local anti-tumor immunity. This approach may stand alone or be combined with other immunotherapies such as immune checkpoint inhibition. Recent progress in tumor-targeting nanoparticles [47], and cellular vehicles such as mesenchymal stem cells [48, 49], will facilitate more precise delivery of these chemokines to the tumor site to generate the chemotactic gradients and local effects.

## Abbreviations

CCK-8: cell counting kit-8
CCL3: C-C motif chemokine ligand 3
CCR1: C-C motif chemokine receptor 1
cDC1: type 1 conventional dendritic cell
cDCs: conventional dendritic cells
CRC: colorectal cancer
DCs: dendritic cells
DMEM: Dulbecco’s Modified Eagle Medium
FACS: fluorescence-activated cell sorting
FBS: fetal bovine serum
ITGAX: integrin alpha X
MHC: major histocompatibility complex
mRNA: messenger RNA
NK: natural killer
PBS: phosphate-buffered saline
PCR: polymerase chain reaction
Th1: T helper 1
TME: tumor microenvironment
Treg: regulatory T
XCL1: X-C motif chemokine ligand 1

## Declarations

### Copyright

© The Author(s) 2022.
